# Repayment policy for multiple loans

**DOI:** 10.1371/journal.pone.0175782

**Published:** 2017-04-21

**Authors:** Yasmin Agueda Rios-Solis, Mario Alberto Saucedo-Espinosa, Gabriel Arturo Caballero-Robledo

**Affiliations:** 1 Systems Engineering, Universidad Autónoma de Nuevo León, Monterrey, Nuevo León, Mexico; 2 Rochester Institute of Technology, Microsystems Engineering Department, Rochester, United States of America; 3 CINVESTAV-Monterrey, Monterrey, Nuevo León, Mexico; IUMPA - Universitat Politecnica de Valencia, SPAIN

## Abstract

The Repayment Policy for Multiple Loans is about a given set of loans and a monthly incoming cash flow: what is the best way to allocate the monthly income to repay such loans? In this article, we close the almost 20-year-old open question about how to model the repayment policy for multiple loans problem together with its computational complexity. Thus, we propose a mixed integer linear programming model that establishes an optimal repayment schedule by minimizing the total amount of cash required to repay the loans. We prove that the most employed repayment strategies, such as the *highest interest debt* and the *debt snowball* methods, are not optimal. Experimental results on simulated cases based on real data show that our methodology obtains on average more than 4% of savings, that is, the debtor pays approximately 4% less to the bank or loaner, which is a considerable amount in finances. In certain cases, the debtor can save up to 40%.

## Introduction

A debtor with a set of multiple loans faces the problem of simultaneously repaying them each month: home mortgages, college tuition loans, short-term credit card debts, bank overdrafts, and car loans. That is, the average household must consider different debt instruments that have different starting and ending times, different ordinary and default interest rates, and penalties for not paying its monthly installments.

Given a set of loans and a monthly incoming cash flow, the question this study seeks to address is: what is the best way to allocate the monthly income to repay such loans? This problem becomes even more challenging when income is less than expected, and the debtor faces the problem of deciding which loans to prioritize. Similarly, when income exceeds expectations and there is extra capital available in a particular month, the debtor then faces the task of determining where to allocate this extra money.

This problem is known in the literature as the Repayment Policy for Multiple Loans (RPML). A simplified version of the RPML problem has been previously modeled as a single machine scheduling problem with the objective of minimizing the maximum completion time of the debts, where the principals of the debts increase as functions of their starting times [1, 2]. In the work by [1], the debts are non-preemptive, that is, once debtors start repaying a loan, they cannot stop paying it until it is completely paid, which is an unrealistic assumption. On the other hand, [2] assumed a simplified version of the RPML problem and transformed it into a scheduling problem with preemptive processing times, that is, the debtor can stop repaying a loan and then resume it. The scheduling problem is then approximated with a continuous nonlinear optimization problem. This methodology gives approximate solutions to the simplified version of the multiple loans problem. To the best of our knowledge, these two references are the only ones that attempt to tackle the RPML problem, but the methodology we present in this work is the only one that solves it.

To solve the RPML, we propose a mixed integer linear programming model (MILP) that establishes an optimal repayment schedule by minimizing the total amount of cash required to repay the loans. We prove that the most employed repayment strategies, which are even proposed by top consulting firms [3], are not optimal. These non-optimal strategies include the *highest interest debt* and the *debt snowball* methods. The first strategy requires the debtor to make a list of all its loans, ranked by interest rate in nonincreasing order. The debtor then needs to satisfy the minimum payment of all its loans considering the order of the list. Once all the minimum payments are covered, the remaining extra money is allocated to the loan in the first place of the list. After the first ranked debt has been paid, it is erased from the list. This process continues until the debtor has no more debts. Many financial experts would affirm that the highest interest plan is the best strategy to minimize the total repayment amount. As we will see, our model shows that this is far from true. The debt snowball method is similar to the highest interest debt plan, but loans are ordered with respect to their balance (the amount that the debtor has still to pay to the loaner): the loans with the smaller balances are paid first. Other financial experts argue that, even if the popular debt snowball method does not reduce the total amount paid to the bank, it may have psychological benefits [4].

By proposing a mathematical model for the RPML problem, we close the almost 20 year old open question about the complexity of the RPML which, we find, belongs to the NP-hard complexity class, that is, the time required to solve the problem using any currently known algorithm increases very quickly as the size of the problem grows. Meanwhile the simplified versions of RPML considered in [1] and [2] belongs to the polynomial class, that is, there is an algorithm to efficiently solve this tractable problem. For more details on computational complexity see [5, 6], or [7].

Increasing attention has been devoted to identifying the proper amount of credit a person should be offered, so his/her probability to be on default is minimum [8–11]. It is important to note that the RPML problem does not plan the loans a debtor should acquire, nor their schedule, as regularly occurs in the cash flows problems, where the aim is to maximize net present value [12–16]. The RPML problem deals, in contrast, with an already fixed set of debts, and aims to minimize the total amount of cash required to repay them.

The rest of this paper is organized as follows. In the next section, we present a mixed integer linear programming to solve the RPML problem and show that the problem is NP-hard. Then, we exhibit the performance of the MILP and compare its solutions with the most used financial methods that attempt to solve the RPML. A final section concludes this work.

## Materials and methods

### Mixed integer linear programming (MILP) for the RPML problem

Let *L* = {1,…,*n*} be the set of *n* loans to be considered. Each loan *j* is composed by a principal amount *m*_*j*_, a release time *r*_*j*_ corresponding to the month in which debt *j* is acquired, a due time *d*_*j*_ when the debt would be *ideally* repaid, and an ordinary monthly interest rate $i_{j}^{t}$ in a month *t*. Indeed, we can consider a loan that has a variable interest rate. Suppose that the debtor disposes of a maximum amount of money *f*^*t*^ in a month *t* to pay the loans. We let this amount to differ for each period to account for an extra or lesser income, such as a Christmas bonus.

Some debt instruments, such as credit cards, have *minimum payments* every month. The debtor can choose to pay less than this minimum at the expense of obtaining a default interest rate $h_{j}^{t}>i_{j}^{t}$. The minimum repayment for credit cards that is usually established by banks corresponds to a percentage *pm*_*j*_ of the balance of the loan. Similarly, certain debt instruments impose a penalty *pc*_*j*_ when the debtor repays more than the initially established installment $E_{j}^{t}$ set by the moneylender.

Let us now introduce the variables of the MILP for the RPML problem. The main decision variables are $X_{j}^{t}$, which represent the amount of money to be paid to loan *j* in month *t*. Let $C_{j}^{t}$ and $P_{j}^{t}$ be the penalties that arise when $X_{j}^{t}$ is less than the minimum payment or more than the established installment, respectively, of the loan *j* at time *t*. Further, let $B_{j}^{t}$ be the balance of loan *j* at time *t*, and consider the possibility that the debtor can make some savings *S*^*t*^ each month. For all debts, we set the binary variables

$$\begin{array}{c} Z_{j}^{t}=\left\{\begin{array}{cc} 1 & \text{if}\;\text{loan}\;j\;\text{has}\;\text{a}\;\text{positive}\;\text{balance}\;\text{at}\;\text{time}\;t,\\ 0 & \text{otherwise}. \end{array}\right. \end{array}$$ (1)

The second set of binary variables is needed for debt instruments that incur a penalty when more than the stipulated installment is repaid:

$$\begin{array}{c} Y_{j}^{t}=\left\{\begin{array}{cc} 1 & \text{if}\;\text{the}\;\text{repayment}\;\text{of}\;\text{loan}\;j\;\text{at}\;t\;\text{is}\;\text{more}\;\text{than}\;\text{the}\;\text{established}\;\text{installment}\;E_{j}^{t},\\ 0 & \text{otherwise}. \end{array}\right. \end{array}$$ (2)

The objective function of the RPML problem is to minimize the total amount of money needed to pay all of the loans:

$$\begin{array}{c} \min\sum_{j=1}^{n}\sum_{t=1}^{T}X_{j}^{t}. \end{array}$$ (3)

Note that if we do not restrict *T*, some loans could, in theory, be paid at an infinite time. To avoid a semi-infinite linear programming [17] we set an upper bound to the repayment time with an arbitrarily large time horizon *T* ≫ max_*j*_(*d*_*j*_) since we have a minimization function.

The first restrictions of the RPML problem indicate that every month *t*, the debtor only has a fixed amount of money *f*^*t*^ available to pay all of its loans together with the saving the debtor has made the previous month:

$$\begin{array}{ccc} \sum_{j=1}^{n}X_{j}^{1}+S^{1} & \leq & f^{1} \end{array}$$ (4)

$$\begin{array}{ccc} \sum_{j=1}^{n}X_{j}^{t}+S^{t} & \leq & f^{t}+S^{t-1},\;t=2,\ldots ,T. \end{array}$$ (5)

We now introduce the financial condition of debt repayment in the MILP, which states that the final value of the debt must be equal to the sum of the final values of the installments. This financial parity is on the basis of the most widely used amortization techniques, which define the equivalent parity involving the present values of the debt and installments. These conditions can be expressed as follows:

$$\begin{array}{c} \sum_{t=r_{j}}^{T}\left(C_{j}^{t}+P_{j}^{t}-X_{j}^{t}\right)\prod_{t^{\prime }=t+1}^{T}\left(1+i_{j}^{t^{\prime }}\right)+m_{j}\prod_{t=r_{j}}^{T}\left(1+i_{j}^{t}\right)=0,\;j\in L. \end{array}$$ (6)

**Fig 1:**
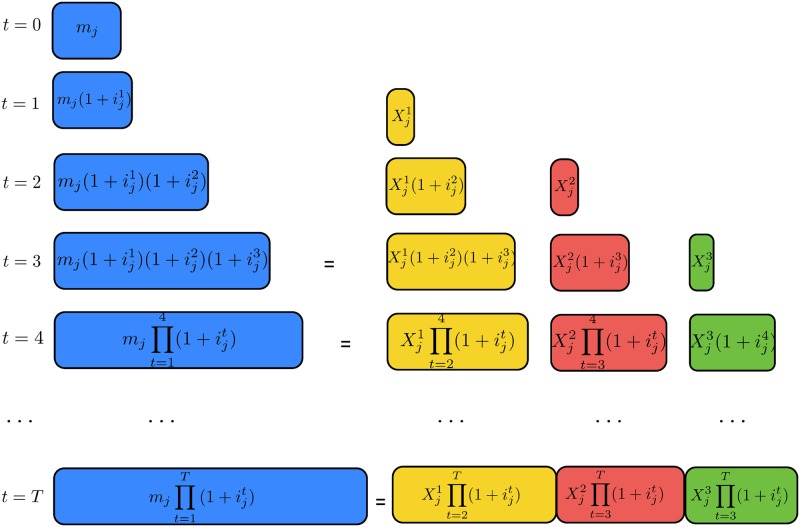
Schematic representation of conditions (6) for a single loan *j*. The principal amount *m*_*j*_ and each installment $X_{j}^{t}$ are multiplied by their accumulation factor after *t* periods. If the installment amounts (yellow, red, and green, respectively) are equal to the principal with its accumulation factor (blue) as it happens at time *t* = 3, then loan *j* has been completely repaid.

Fig 1 illustrates conditions (6) for a single loan *j*. The principal amount *m*_*j*_ is multiplied by its accumulation factor, which gives its amount at compound interest after *t* periods. Similarly, each installment $X_{j}^{t}$ is multiplied by its accumulation factor. If the installment amounts (yellow, red, and green, respectively) are equal to the principal with its accumulation factor (blue) as it happens at time *t* = 3, then loan *j* has been completely repaid. The fact that this equality also holds for *t* = 4, *t* = 5,…, up to time *T*, and that it is coupled with a minimization objective function, allows us to obviate the need to know the *real* completion times of the loans beforehand.

The following restrictions define the balance $B_{j}^{t}$ of loan *j* at time *t*. These equations allow us to know the amount of money that the debtor owes at every time *t* and for each loan *j*:

$$\begin{array}{c} B_{j}^{r_{j}}=m_{j},\;\forall j\in L, \end{array}$$ (7)

$$\begin{array}{c} B_{j}^{t}=\sum_{r_{j}\leq t^{\prime }\leq t-1}\left(C_{j}^{t^{\prime }}+P_{j}^{t^{\prime }}-X_{j}^{t^{\prime }}\right)\prod_{t^{\prime }+1\leq t^{\prime \prime }\leq t}\left(1+i_{j}^{t^{\prime \prime }}\right)+m_{j}\prod_{r_{j}\leq t^{\prime }\leq t}\left(1+i_{j}^{t^{\prime }}\right),\\ \;\;\;\;\;\;\;j\in L,r_{j}<t\leq T. \end{array}$$ (8)

Let *M* be a very large constant that make variables $Z_{j}^{t}=1$ if loan *j* has a positive balance at time *t*:

$$\begin{array}{c} B_{j}^{t-1}\left(1+i_{j}^{t}\right)\leq MZ_{j}^{t},\;j\in L,r_{j}<t\leq T. \end{array}$$ (9)

If the debt is not a credit card debt, then an established installment $E_{j}^{t}$ has to be paid each month. If $X_{j}^{t}$ is not sufficient to cover this installment then variable $C_{j}^{t}$ registers the penalty to be paid:

$$\begin{array}{c} \left(Z_{j}^{t}E_{j}^{t}-X_{j}^{t}\right)\left(1+h_{j}^{t}\right)\leq C_{j}^{t},\;r_{j}<t\leq T. \end{array}$$ (10)

For credit card debts, the minimum payment is a percentage *pm*_*j*_ of the balance $B_{j}^{t}$, and the penalization $C_{j}^{t}$ is computed as follows:

$$\begin{array}{c} \left(pm_{j}B_{j}^{t-1}\left(1+i_{j}^{t}\right)-X_{j}^{t}\right)\left(1+h_{j}^{t}\right)\leq C_{j}^{t},\;r_{j}<t\leq T. \end{array}$$ (11)

Moreover, penalties can also apply if the debtor repays more than the monthly installment. In this case, the penalty is usually a fixed amount *pc*_*j*_. The binary variables defined in Eq (2) determine if the penalty $P_{j}^{t}$ applies, in which case they are also handled through an inequality using the constant *M*:

$$\begin{array}{c} X_{j}^{t}-Z_{t}^{j}E_{j}^{t}\leq Y_{j}^{t}M,\;P_{j}^{t}=Y_{j}^{t}pc_{j},\;j\in L,r_{j}<t\leq T. \end{array}$$ (12)

Notice that our model computes a penalty in the last month that a loan is paid if the debtor pays less than the established installment. This situation can be easily avoided by introducing additional restrictions to the MILP of the RPML problem. Note that credit cards do not face this problem. Finally, we set the nature of the variables:

$$\begin{array}{ccc} & X_{j}^{t}=C_{j}^{t}=P_{j}^{t}=0 & \;\;\;\;\;j\in L,t<r_{j}, \end{array}$$ (13)

$$\begin{array}{ccc} & X_{j}^{t},C_{j}^{t},P_{j}^{t},B_{j}^{t}\geq 0 & \;\;\;\;\;j\in L,t=0,\ldots ,T, \end{array}$$ (14)

$$\begin{array}{ccc} & Z_{j}^{t},Y_{j}^{t}\in \left\{0,1\right\} & \;\;\;\;\;j\in L,t=0,\ldots ,T. \end{array}$$ (15)

The MILP of the RPML problem is composed using the objective function (3) and restrictions Eqs (4)–(15).

Let us now show that the RPML problem can be classified into the NP-hard complexity class by giving the main component needed for a complexity proof by reduction in an intuitive manner. When Eq (8) are substituted in Eq (11), we obtain the following restrictions for *j* ∈ *L*, *r*_*j*_ < *t* ≤ *T*:

$$\begin{array}{c} \left[\sum_{r_{j}\leq t^{\prime }\leq t-2}\left(C_{j}^{t^{\prime }}+P_{j}^{t^{\prime }}-X_{j}^{t^{\prime }}\right)\prod_{t^{\prime }+1\leq t^{\prime \prime }\leq t}\left(1+i_{j}^{t^{\prime \prime }}\right)\right]-MZ_{j}^{t-1}\leq -m_{j}\prod_{r_{j}\leq t^{\prime }\leq t}\left(1+i_{j}^{t^{\prime }}\right). \end{array}$$ (16)

Since the right-hand side of Eq (16) is a constant and variables $Z_{j}^{t}$ are binary, these equations correspond to the restrictions of the multiple knapsack problem, which is an NP-hard problem [18, 19]. Then, the RPML problem inherits its complexity from the multiple knapsack problem.

The simplified version of the RPML problem considered in references [1] and [2] would correspond to a linear programming with the same objective function (3), but considering only restrictions Eqs (4) and (6). Notice that the only variables needed in this case are positive real variables. While the RPML problem is an NP-hard problem that may take an exponential time to be solved, this simplified version belongs to the polynomial complexity class.

The MILP for the RPML problem can be solved using a branch-and-bound algorithm [20], as shown in Results and discussion section, and is elegant enough to obtain high-quality solutions for real household size instances in a reasonable time.

Note that the RPML methodology provides the debtor with the optimal plan to minimize the total amount of money, including default rates and penalties to be paid for the entire year. If her income changes or if interest rates are not as expected, the debtor has to compute the RPML methodology again to obtain a new plan.

## Results and discussion

The MILP we have presented is the only methodology that solves the RPML problem. This section provides experimental results to indicate the efficiency of the RPML formulation in terms of the size of the instances. Moreover, we prove that the most popular repayment strategies such as the *highest interest debt* and the *debt snowball* methods are not optimal.

### Instances

Many types of debts with different legal and financial characteristics exist. In this work, we chose the main four types of debts that a household may face: cars, houses, credit cards, and bank loans. To this end, 550 instances were generated: 110 with four debts (e.g., one car, one house, one credit card loan, and one bank loan, or two cars and two houses, etc.), 220 with eight debts, and 220 with 12 debts. All instances, their solutions, and times can be found in the following repository: https://doi.org/10.6084/m9.figshare.4823518.v1.

Each instance was randomly generated by uniformly choosing its parameters from the intervals presented in Table 1 (partially based on [21]). The first three lines correspond to the principal, annual interest and annual default interest, respectively. The fourth line is the percentage of the loan balance considered to compute the minimum payment (only for credit cards), while the fifth line is the penalty incurred when the debtor pays more than the stipulated installment. The sixth line shows that the debts were acquired in the same month. Having different release dates for the debts makes the instances easier to solve by our methodology since less variables are active at the same period of time. Finally, the last line shows the ideal ending times of the loans.

**Table 1:** Intervals from which the parameters of the instances were uniformly chosen.

	cars	houses	credit cards	bank loans
principal *m*_*j*_	[100000,700000]	[750000,4000000]	[100,400000]	[1000,300000]
interest $i_{j}^{t}$	[0.07,0.17]	[0.06,0.2]	[0.06,0.4]	[0.06,0.5]
default int. $h_{j}^{t}$	[0.17,0.27]	[0.16,0.3]	[0.16,0.5]	[0.16,0.6]
min. % *pm*_*j*_	-	-	[0.05,0.1]	-
> installment *pc*_*j*_	[500,1000]	[1000,2000]	-	-
release dates *r*_*j*_	0	0	0	0
final month *d*_*j*_	[6,54]	[12,252]	[3,51]	[3,60]

The stipulated installments *E*_*j*_ were computed using a simple amortization formula. The monthly incomes *f*^*t*^ of the debtors were obtained by adding all the installments they should pay monthly and multiply this value by a random number between [-0.5,0.5].

In many cases, the consequences of neglecting the dates of the payments are more serious than a penalty to pay. For example when you buy a car or a house, if you do not pay a certain number of installments or you pay too little, you risk losing everything. While these type of legal restrictions are not considered in our instances nor in the model for the RPML, they could be easily adapted to take them into account.

### Algorithms

Four algorithms are tested in this section.

1. RPML: The MILP of the RPML problem is solved using the integer linear solver Gurobi [22] which is based on a branch-and-bound algorithm. Since this is an exhaustive search of the solution space, it can take a long time to find the optimal value. However, a time limit of 30 minutes results in a high quality solution with a Gap that indicates the percentage deviation from the optimum solution (when the Gap is closer to 0, then the solution value is closer to the optimum). The Gap is computed as (ObjBound-ObjVal)/ObjVal, where ObjBound and ObjVal are the MILP objective bound and incumbent solution objective, respectively, as obtained from the branch-and-bound algorithm [22].
2. HInterest: the highest interest plan favors the loans with highest interest rates.
3. Snow: the snowball plan, where the loans with minimum balance are favored.
4. Ave: We present a new rule of thumb similar to the highest interest plan, but where loans are ordered with respect to the average value of the ordinary and default interests. We name this strategy as the *Debt Average* plan.

The RPML model was coded in C++ and solved using Gurobi 6.04 on a Mac Pro 3.5 GHz 6-Core Intel Xeon E5 with 32 RAM. We used the default Gurobi settings except for the time limit and the optimality gap which were set to 30 minutes and 0.000001, respectively.

Algorithm 1 presents the pseudo code of the highest interest plan, which has been implemented in C++ and takes less than one second for each instance. The first repeat loop is to pay as many minimum payments (or installments) as possible. The while loop is for allocating extra money to the loans with highest interests. The same algorithm is used for both the debt snowball and the average plan. The only difference is that *L* is arranged in non decreasing order considering the loan balances in the former algorithm, while it is arranged in non increasing order of the average of the normal and default interest rates on the later.

**Algorithm 1** Highest interest rate plan

**Require**: *L* in non increasing order of interest rates; *j* is its first element

1: **repeat**

2: pay min(minimum payment of loan *j*, *f*^*t*^) to loan *j*

3: **if** loan *j* is completely paid **then** remove *j* from *L*

4: *j* is the next element in *L*, update *f*^*t*^

5: **until** *f*^*t*^ = 0 or all minimum payments have been covered

6: set again *j* as the first element in *L*

7: **while** *f*^*t*^ > 0 and *L* is no empty **do**

8: allocate min(*f*^*t*^, balance of *j*)

9: **if** loan *j* is completely paid **then** remove *j* from *L*

10: *j* is the next element in *L*, update *f*^*t*^

11: **end while**

### Experimental results

In Table 2 we present the relative savings when our model is used to solve the RPML problem with respect to the previously mentioned rules of thumb: “HInterest”, “Snow”, and “Ave” (last three columns). In the first column we show the total number of loans, the second column corresponds to the very large time horizon *T*. For each type of instance, we show the average “Gap” and the average “Time” in seconds taken to solve the MILP of the RPML problem. Each line considers the 110 instances with four debts, the 110 instances with eight debts and *T* = 120, the 110 instances with eight debts but with *T* = 240, and the 220 with 12 debts, respectively. The broadly used method accepted by many respectable financial experts is the highest interest rule.

**Table 2:** Relative savings when using our model for the RPML problem with respect to the previously mentioned rules of thumb: “HInterest”, “Snow”, and “Ave”.

Instances		RPLM Performance		Relative savings (%) of RPML vs:		
*L*	*T* (months)	Gap	Time (s)	HInterest	Snow	Ave
4	120	0.00017	27	5.84	7.19	6.64
8	120	0.01129	581	4.62	5.99	5.27
8	240	0.02324	619.6	4.56	7.42	5.58
12	300	0.20677	1057.7	3.43	5.67	4.06
	Average	0.06	571.37	**4.61**	6.57	5.39

By using the RPML methodology we obtain on average more than 4% of savings, that is, the debtor pays approximately 4% less to the bank, which is a considerable amount in finances. This behavior is more dramatic when compared with the debt snowball rule (more than 6%), and the average rule (more than 5%). When the RPML problem is solved with our model, the proximity to the optimum is 0.06 on average, and it is reached in around 600 seconds. Notice that the instances with 12 loans are the hardest to solve with more computational time and higher Gap.

**Fig 2:**
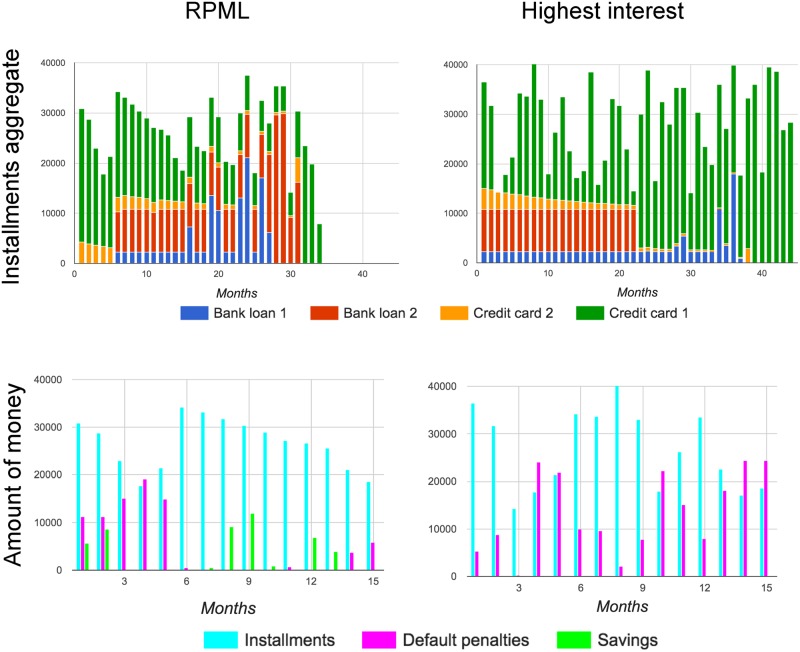
A particular instance that saves 40% of the total repayments by using RPML instead of the highest interest plan. The upper plots represent the installments aggregate to the different loans each month. The bottom plots show the total installments, the amount of default penalties generated that month, and the monthly cash savings $S_{j}^{t}$.

In Fig 2 we present a particular instance that would save 40% of the total repayments by using RPML instead of the highest interest plan. This improvement is the best one obtained from the 550 instances that were tested and, remarkably, it has only two bank loans and two credit card loans. The left side plots of the figure correspond to the RPML method, while the right ones correspond to the highest interest plan. The upper plots represent the installments aggregate to the different loans each month. The order of the loans with respect to decreasing interest rate is bank loan 1, bank loan 2, credit card loan 2, and credit card loan 1. The bottom plots in Fig 2 show the monthly installments, the amount of default penalties generated each month, and the monthly cash savings $S_{j}^{t}$. Both methods perform very differently. Indeed, the RPML ends its repayments in month 34 while the highest interest plan finishes in month 45. In the RPML, note that the two credit cards which are the ones with smallest interest rates are the ones favored the first months, even if bank loan 2 is the one with the highest default rate. We can see in the bottom graph corresponding to the RPML method a counterintuitive behavior: in the first months, the RPML model proposes to save money even if the debtor generates default penalties that might be even greater than the amount of the installments (as in month 4). However, these savings will palliate the lack of cash that the debtor will face during months 3, 4 and 5, and in this manner, the default penalties of these months are less marked, as it can be seen when comparing with the highest interest plot. The cyclic behavior of the savings proposed by the RPML plan is the powerful arm that allows reducing the total amount of repayments by diminishing the default penalties.

In Tables 3 and 4, we analyze the behavior of the RPML formulation by examining the type of loan that makes the instance to be more difficult to solve by the RPML methodology. In Table 3 all the 550 instances are ordered with respect to the number of house-related debts (first column). For example, the first line shows the average of all the instances without house loans, independently of other parameters like the horizon time *T*. The other columns correspond to the average Gap, the average time in seconds, the average benefit of solving the instance with the RPLM methodology instead of using the highest interest, the snowball, and the average rules, respectively. In Table 4 we show the same information as in Table 3; however, all the 550 instances are ordered with respect to the number of credit-card related debts (first column).

**Table 3:** Behavior of the RPML formulation: instances are ordered with respect to the number of house-related debts (“Houses”).

Houses	Gap	Time	HInterest	Snow	Ave
0	0.003	175.9	4.6	6.4	5.3
2	0.018	616.1	4.3	4.9	4.6
4	0.078	1249.1	4	5.5	4.7
8	0.287	1719.4	4.1	9.1	5.9
12	2.282	1800.1	3.7	16.3	6.7

**Table 4:** Behavior of the RPML formulation: instances are ordered with respect to the number of credit-card debts (“CC”).

CC	Gap	Time	HInterest	Snow	Ave
0	0.164	792.6	5.5	8	6.6
2	0.030	822.8	4.1	5.2	4.1
4	0.015	522.8	3.3	5.2	4
8	0.002	161.2	2.1	3.5	2.3
12	0	2.1	0.5	2.3	1

Table 3 indicates that a higher number of home loans results in the most difficult instances because the gap and the solving times are larger. Indeed, house-related debts are usually long-term obligations that are translated as more variables in the RPML model.

The opposite behavior occurs in Table 4 with credit cards, that is, the RPML formulation performs better when the instances have more credit cards because they are short-term debts and many of the binary variables in the RPML model are zero. Nevertheless, the benefit of using the RPML methodology with instances with more credit-card debts is reduced with respect to the other alternatives. Indeed, the benefit is around 0.5% with respect to the highest interest rule.

**Fig 3:**
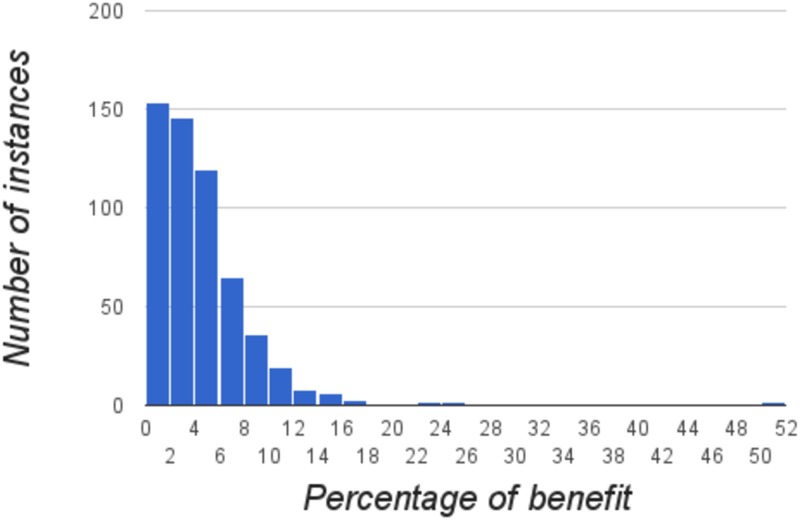
Histogram with the number of instances which have a particular relative saving, in percentage, when solved with the RPML formulation instead of being planned with the highest interest rule.

Fig 3 shows a histogram of the number of instances that have a particular relative saving, in percentage, when solved using our model for the RPML problem instead of being planned with the highest interest rule. We notice that many instances exist for which the benefit is between 0 and 3%, although even a 1% of the benefit in a million dollar loan is already relevant. Such instances have more credit card debts. Importantly, there are also many instances for which the improvement is more than 3% and up to 25% for some of them.

## Conclusion

This work presents a linear programming formulation to determine the optimal repayment policy for multiple loans. Using such a formulation shows that the repayment policy for multiple loans belongs to the NP-hard complexity class, whereas the simpler versions considered in the literature can be solved in polynomial time.

The experimental results indicate that the proposed exact-based methodology gives, in a reasonable amount of time, high-quality solutions for real size instances. A comparison with other approaches that act as “rules of thumb” demonstrates the complexity of estimating the optimal repayment policy for multiple loans, which evidences the great relevance of the formulation proposed in this work to solve the problem.

To formulate the RPML problem we focused on generating a MILP for which even difficult instances can be solved almost to optimality in less than 30 minutes.

The RPML formulation can be enhanced if the MILP considers the stochastic behavior of interest rates or in the monthly amount used to repay the debts. Moreover, the RPML problem must be adapted to enterprise cases in which other types of debts are considered such as government bonuses or financial stocks.
